# Maltose Processing and Not β-Amylase Activity Curtails Hydrolytic Starch Degradation in the CAM Orchid *Phalaenopsis*

**DOI:** 10.3389/fpls.2019.01386

**Published:** 2019-11-14

**Authors:** Nathalie Ceusters, Mario Frans, Wim Van den Ende, Johan Ceusters

**Affiliations:** ^1^KU Leuven, Department of Biosystems, Division of Crop Biotechnics, Research Group for Sustainable Crop Production & Protection, Campus Geel, Geel, Belgium; ^2^KU Leuven, Department of Biology, Laboratory of Molecular Plant Biology, Leuven, Belgium; ^3^UHasselt, Centre for Environmental Sciences, Environmental Biology, Diepenbeek, Belgium

**Keywords:** hydrolytic starch degradation, phosphorolytic starch degradation, DPE2, maltase, crassulacean acid metabolism, *Phalaenopsis*

## Abstract

Crassulacean acid metabolism (CAM) is one of the three photosynthetic pathways in higher plants and is characterized by high water use efficiency. This mainly relies on major nocturnal CO_2_ fixation sustained by degradation of storage carbohydrate such as starch to provide phosphoenolpyruvate (PEP) and energy. In contrast to C3 plants where starch is mainly degraded by the hydrolytic route, different observations suggested the phosphorolytic route to be a major pathway for starch degradation in CAM plants. To elucidate the interplay and relevant contributions of the phosphorolytic and hydrolytic pathways for starch degradation in CAM, we assessed diel patterns for metabolites and enzymes implicated in both the hydrolytic route (β-amylase, DPE1, DPE2, maltase) and the phosphorolytic route (starch phosphorylase) of starch degradation in the CAM orchid *Phalaenopsis* “Edessa.” By comparing the catalytic enzyme activities and starch degradation rates, we showed that the phosphorolytic pathway is the major route to accommodate nocturnal starch degradation and that measured activities of starch phosphorylase perfectly matched calculated starch degradation rates in order to avoid premature exhaustion of starch reserves before dawn. The hydrolytic pathway seemed hampered in starch processing not by β-amylase but through insufficient catalytic capacity of both DPE2 and maltase. These considerations were further corroborated by measurements of enzyme activities in the CAM model plant *Kalanchoë fedtschenkoi* and strongly contradict with the situation in the C3 plant Arabidopsis. The data support the view that the phosphorolytic pathway might be the main route of starch degradation in CAM to provide substrate for PEP with additional hydrolytic starch breakdown to accommodate mainly sucrose synthesis.

## Introduction

Crassulacean acid metabolism (CAM) is one of the three photosynthetic pathways present in higher plants and is characterized by an optimized water use efficiency (WUE) by taking up CO_2_ predominantly at night when evapotranspiration rates are low. It is convenient to recognize four distinct phases of gas exchange in CAM plants, which are also used to describe the photosynthetic performance. Sequestration of CO_2_ at night (Phase I) occurs *via* the enzyme phosphoenolpyruvate carboxylase (PEPC) with the 3-C substrate phosphoenolpyruvate (PEP) provided by the glycolytic breakdown of carbohydrate. The final 4-C product, malic acid, is stored in a large central vacuole. At the start of the next day, in Phase II, stomata will gradually close and external CO_2_ is mainly fixed by ribulose-1,5-bisphosphate carboxylase-oxygenase (rubisco). Gas exchange is curtailed by stomatal closure during the middle of the day (Phase III), thereby reducing transpirational water losses and improving WUE. During this phase, malic acid exits the vacuole and decarboxylation releases CO_2_ which is re-fixed by rubisco. In Phase IV stomata open again towards the end of the day and external CO_2_ is mainly sequestered *via* rubisco (Osmond, 1978).

Because of the tight relationship between PEP availability and nocturnal CO_2_ fixation (Dodd et al., 2003; Ceusters et al., 2019), the provision of carbon skeletons for the synthesis of PEP represents a significant sink for carbohydrate. Besides starch, also glycolysis driven by sucrose hydrolysis can attribute to PEP formation. Various studies with CAM bromeliads showed diurnal accumulation of both starch and soluble sugars followed by nocturnal decrease to fuel the dark reactions of CAM (Christopher and Holtum, 1998; Popp et al., 2003; Ceusters et al., 2008; Ceusters et al., 2009a; Ceusters et al., 2009b; Ceusters et al., 2011; Ceusters et al., 2014). Therefore, transitory starch plays a crucial role in many CAM plants. In contradiction to the starch biosynthesis pathway for which the major steps have been well characterized, the process of transitory starch breakdown in CAM has not yet been elucidated (Lloyd and Kossmann, 2015). In starch storing CAM species different modes of starch degradation might be deployed for nocturnal starch breakdown, i.e. the phosphorolytic and/or the hydrolytic route (Borland et al., 2016; Brilhaus et al., 2016). Both routes are assumed to share the same enzymes to induce the initial attack to starch granules. Glucan water dikinase (GWD) initially phosphorylates the starch granule which consists of branched amylopectin and linear amylose. Debranching enzymes limit dextrinase (LDA) and isoamylase (ISA) bring about cleavage of the branch points in amylopectin. Different enzymes are subsequently used for further processing of linear amylose into soluble sugars (Weise et al., 2011). Whilst phosphorolytic starch degradation in the chloroplast mainly delivers glucose-6-phosphate (Glc6P) for export, the export product resulting from the hydrolytic starch breakdown is maltose (Kore-eda and Kanai, 1997; Weise et al., 2004).

Several observations indicate the phosphorolytic pathway to be the main route in CAM. The facultative CAM plant *Mesembryanthemum crystallinum* (common ice plant) is able to switch from C3 to CAM under drought or salt stress and has emerged as a useful model for exploring CAM. By shifting to the CAM mode, a considerable export of Glc6P out of the chloroplast was observed whilst maltose export dramatically diminished (Neuhaus and Schulte, 1996). In addition, consistent increases in transcript abundances and in the activity of enzymes involved in the phosphorolytic route were observed when switching to CAM mode (Paul et al., 1993; Dodd et al., 2003; Cushman et al., 2008; Taybi et al., 2017). Phosphorolytic degradation of starch is mediated *via* starch phosphorylase, which releases glucose 1-phosphate (Glc1P) from the non-reducing ends of glucan chains. Subsequently, phosphoglucomutase (PGM) catalyses the conversion of Glc1P to Glc6P which is exported to the cytosol *via* the plastidic Glucose 6-Phosphate: Phosphate Translocator (GPT). GPT transcript abundance has been found to show a > 70-fold upregulation in *M. crystallinum* upon CAM induction (Häusler et al., 2000; Cushman et al., 2008; Kore-eda et al., 2013). In C3 plants the phosphorolytic mode of starch degradation has been proposed to provide carbon solely for metabolism inside the chloroplast *via* the pentose phosphate pathway, particularly under stress conditions when photorespiration is elevated (Weise et al., 2006; Zeeman et al., 2007).

The hydrolytic route of starch degradation is mainly mediated *via* β-amylase, leading to the production of maltose. Besides maltose, a range of short malto-oligosaccharides will emerge as by-products. These require further processing by chloroplastic glucanotransferase disproportionating enzyme (DPE1) to yield glucose and a spectrum of larger, linear oligosaccharides which can further be metabolized again by β-amylase. Notwithstanding α-amylase also possesses the capacity to degrade transitory starch, no phenotype has been detected in knockout mutants of Arabidopsis (Yu et al., 2005). To reach the cytosol glucose and maltose require the dedicated transporters Maltose Excess Protein1 (MEX1) and Plastidic Glucose Transporter (pGlcT) (Niittylä et al., 2004; Smith et al., 2005; Cho et al., 2011). In Arabidopsis, the major export product out of the chloroplast has been found to be maltose and further processing in the cytosol requires a cytosolic disproportionating enzyme (DPE2), which transfers one glucose unit from maltose to an acceptor (a form of cytosolic heteroglycan) and releases the other glucose molecule (Lu and Sharkey, 2004). Based on calculations of starch breakdown in Arabidopsis wild types and DPE2 mutants, direct hydrolysis by maltase (α-glucosidase) has been considered of minor importance (Chia et al., 2004). The potential contribution of maltase in CAM plants is yet unknown. In the cytosol, cytosolic hexokinase catalyses the conversion from glucose to Glc6P, which together with the Glc6P derived from the phosphorolytic degradation, can be used for further sucrose synthesis (Borland et al., 2016).

Several studies on the common ice plant (*M. crystallinum*) demonstrated the upregulation of transcript abundances and activities of a range of starch-degrading enzymes implicated in both the hydrolytic and phosphorolytic routes of starch degradation (α-amylase, β-amylase, starch phosphorylase, and disproportionating enzyme (DPE1 and DPE2)) following CAM induction (Paul et al., 1993; Neuhaus and Schulte, 1996; Häusler et al., 2000; Dodd et al., 2003; Cushman et al., 2008). These observations pose important questions about 1) the relative contribution of both routes of starch degradation in CAM and 2) whether specific enzymes might be rate limiting in either phosphorolytic and hydrolytic pathways. Exploiting the phosphorolytic pathway might confer specific advantages for CAM plants as Glc6P not only functions as an allosteric activator for PEPC but its glycolytic conversion in the cytosol also provides ATP, which adds in energizing nocturnal accumulation of malate in the vacuole (Holtum et al., 2005; Weise et al., 2011; Cheung et al., 2014). However, it has recently been postulated that the use of both the hydrolytic and phosphorolytic starch degradation pathway in CAM plants might provide a means to accommodate carbohydrate partitioning between competing forces of growth and nocturnal carboxylation during the diel cycle (Borland et al., 2016). To elucidate the interplay and relevant contributions of the phosphorolytic and hydrolytic pathways for starch degradation we assessed diel *in vitro* activity patterns for enzymes implicated in either the hydrolytic route (β-amylase, DPE1, DPE2, maltase) or the phosphorolytic route (starch-phosphorylase) of starch degradation in the CAM orchid *Phalaenopsis* “Edessa.” These analyses were further complemented with measurements of diel leaf gas exchange and important metabolite dynamics (starch, malic acid, sucrose, glucose, fructose, maltose, Glc6P, Glc1P). By comparing *in vitro* catalytic activities and starch degradation rates we were able to gain more insight about the relative contributions of both pathways and postulated a bottleneck limiting hydrolytic starch conversion.

## Materials and Methods

### Plant Material and Sampling

*Phalaenopsis* “Edessa” is an obligate starch-storing CAM plant and belongs to the family of the Orchidaceae. In starch-storing CAM plants starch is the main carbohydrate to accommodate nocturnal carboxylation whilst soluble sugar storing species employ sucrose, glucose, and/or fructose. Vegetative plants of 16 weeks old were cultivated in a growth room with a constant temperature of 28°C, a relative humidity of 75% and a 12h photoperiod (zeitgeber time ZT0-ZT12) with photosynthetic photon flux density (PPFD) of 100 µmol m^-2^ s^-1^. Watering was performed twice a week; once with a nutrient solution Peters 20N-8.7P-16.6K of 1 mS cm^-1^ and once with water. After six weeks, leaf samples (n = 5) were taken from the upper one-third of young fully expanded source leaves during a cycle of 24 h starting from 08.00 h (ZT0) every 2 h until 08.00 h the next morning (ZT24). The samples from 08.00 h (ZT0 and ZT24) were taken when the lights were turned on whilst the samples taken at 20.00 h (ZT12) were taken in the dark under green safety light.

At ZT12 samples were also taken from leaf pair six of the obligate model CAM plant *Kalanchoë fedtschenkoi* in order to provide comparison with the *Phalaenopsis* enzyme activity determinations. These plants were also grown in a growth room with a temperature of 25 °C and 19 °C during the light and dark periods respectively, with a relative humidity of 75% and a 12-h photoperiod (zeitgeber time ZT0-ZT12) with photosynthetic photon flux density (PPFD) of ∼250 µmol m^-2^ s^-1^. Plants were grown under these conditions for at least 8-10 weeks before sampling at which time all plants had around 10 to 12 leaf pairs.

All samples were immediately frozen in liquid nitrogen, powdered and stored at -80°C.

### Gas Exchange Measurements

Net CO_2_ exchange was measured on the youngest fully expanded leaves, using a LCi Portable Photosynthesis System (ADC BioScientific Ltd., UK; https://www.adc.co.uk/). The top part of the leaf was enclosed in a broad leaf chamber (6.25 cm2) and the incoming air was passed through a 20-l bottle to buffer short-term fluctuations in the CO_2_ concentration. After six weeks, gas exchange data were collected over a 24-h period with measurements obtained at 15-min intervals (n = 3).

### Chemical Analyses of Metabolites

Soluble sugars (glucose, fructose, sucrose, and maltose) were extracted using hot water (80°C), as described by Tarkowski et al. (2019), and quantified by high performance anion exchange chromatography with pulsed amperometric detection, as described by Verspreet et al. (2013).

Extraction for measurements of starch, malic acid, and phosphorylated sugars (Glc6P and Glc1P) was performed as described by Chen et al. (2002), but with modifications. Approximately 180 mg of powdered tissue was mixed with 450 µl of ice-cold 4% (v/v) HClO_4_. The mixture was allowed to thaw slowly on ice for 30 min. The resulting suspension was then centrifuged at 4°C for 10 min at 16,200 g. The insoluble residue from the perchloric acid extraction was used to determine starch content spectrophotometrically at 340 nm as glucose equivalents (Genesys 10S UV-VIS, Thermo Scientific, USA; https://www.thermofisher.com), following digestion with a mix of amyloglucosidase (EC 3.2.1.3) and α-amylase (EC 3.2.1.1). The analyses were conducted as earlier described by Ceusters et al. (2008). The supernatant from the HClO_4_ extraction was neutralized at 4°C with 5 M K_2_CO_3_, and the resulting potassium perchlorate precipitate was removed by 10 min centrifugation at 16,200 g and 4°C. Five mg activated charcoal was added to the supernatant, and after 15 min at 4°C, removed by new centrifugation. The supernatant was used for measurement of malic acid, Glc6P, and Glc1P. Malic acid was measured in a 500-µl reaction mixture (EnzytecTM code n° E1215) containing: glycylglycinebuffer, NAD, glutamate oxaloacetate transaminase (GOT, EC 2.6.1.1). Analysis was performed spetrophotometrically by determining the change in absorbance at 340 nm after adding L-malate dehydrogenase (L-MDH, EC 1.1.1.37). The phosphorylated sugars Glc6P and Glc1P were measured in a 500-µl reaction mixture containing: 100 mM HEPES-KOH, pH 7.6, 4 mM MgCl_2_, 0.2 mM NADP and 1 unit glucose-6-phosphate dehydrogenase (G6PDH, EC 1.1.1.49) for Glc6P, then 1 unit glucose phosphate isomerase (GPI, EC 5.3.1.9) and 1 unit PGM (EC 5.4.2.2) for Glc1P (Mohanty et al., 1993). Analysis was performed spectrophotometrically by determining the change in absorbance at 340 nm.

### Enzyme Activities

All extraction steps were performed by homogenizing leaf material at 4°C in extraction buffer containing: 0.3 M N-(2-hydroxyethyl)piperazine-N′-(2-ethane sulphonic acid) (HEPES, pH 7.4), 20 mM MgCl_2_, 1 mM EDTA, 1 mM EGTA, 4.6 mM DTT, 80 mM benzamidine, 1 mM phenylmethylsulfonyl fluoride (PMSF), and 0.1 mM Triton X-100.

The extraction of β-amylase was based on the method described by Zeeman et al. (1998), but with modifications. After extraction, the homogenate was centrifuged at 4°C for 10 min at 16,200 g. The supernatant was incubated at 30˚C for 30 min in a 250 µl reaction mixture containing 50 mM 3-(N-Morpholino) propanesulfonic acid (MOPS, pH 6.4), 5 mM EDTA, 5 mM DTT, and 15 mg ml–1 soluble potato starch. After incubation, the reaction was stopped by boiling for 5 min and maltose was hydrolyzed by incubation in a 135 µl reaction mixture, containing 74 mM 2-(N-morpholino) ethanesulfonic acid (MES, pH 6.8), and 2 units maltase (EC 3.2.1.20), for 2 h at 37˚C. The reaction was stopped by boiling for 5 min and the resulting glucose was determined in a 500 µl reaction mixture containing: 25 mM HEPES (pH 7.9), 1 mM MgCl_2_, 1.2 mg ml^-1^ ATP, 1.0 mg ml^-1^ NAD, and 2 units hexokinase (EC 2.7.1.1). Analysis was performed spectrophotometrically (Genesys 10S UV-VIS, Thermo Scientific, USA) by determining the change in absorbance at 340 nm after adding 4 units G6PDH.

The extraction of maltase (α-glucosidase) and plastidic D-enzyme (4-α-glucanotransferase: DPE1) was based on the methods described by Kruger and ap Rees (1983) and by Takaha et al. (1993) respectively, but with modifications. After extraction, the homogenate was centrifuged at 4°C for 10 min at 16,200 g. The supernatant was incubated at 30˚C for 30 min in a 250 µl reaction mixture containing 50 mM sodium acetate (pH 5.2), 90 mM maltose for maltase and 50 mM MOPS-NaOH (pH 6.8), and 60 mM maltotriose for plastidic D-enzyme. The reaction was stopped by boiling for 1 min and 100 µl of pure extract was also boiled for 1 min as control. Subsequently glucose was determined in a 500 µl reaction mixture containing: 25 mM HEPES (pH 7.9), 1 mM MgCl_2_, 1.2 mg ml^-1^ ATP, 1.0 mg ml^-1^ NAD and 2 units hexokinase (EC 2.7.1.1). Analysis was performed spectrophotometrically by determining the change in absorbance at 340 nm after adding 4 units G6PDH.

The extraction and assay of cytosolic D-enzyme (DPE2) was based on the method described by Chia et al. (2004), but with modifications. After extraction, the homogenate was centrifuged at 4°C for 10 min at 16,200 g. Subsequently the supernatant was desalted by passing twice through a 0.5-ml column of Sephadex G-25, equilibrated with 100 mM Tris-HCl (pH 7.5 at 4°C), 1 mM DTT, 1 mM benzamidine, and 5% (w/v) glycerol. Three series of the same samples were incubated at 30°C for 2 h. The first series for assay of glucose production contained: 31 mM MOPS (pH 7), 10% (v/v) glycerol, 2.5% (w/v) oyster glycogen, and 30 mM maltose in a 200 µl reaction mixture. The two remaining series are incubated lacking either maltose or glycogen to exclude glucose not from the reaction catalzsed by DPE2. The reaction was stopped by boiling for 2 min and analysis was performed spectrophotometrically by determining the change in absorbance at 340 nm after adding 4 units G6PDH. The activity was calculated as the difference between the amount of glucose produced in the first incubation series, and the sum of the amounts of glucose produced in the two-control series.

The extraction of starch phosphorylase (α-glucan phosphorylase) was based on the method described by Kumar and Sanwal (1982), but with modifications. After extraction, the homogenate was centrifuged at 4°C for 10 min at 16,200 g. The supernatant was incubated at 30°C for 30 min in a 200 µl reaction mixture containing 0.2 M MOPS (pH 7.1), 15 mg ml^-1^ soluble potato starch, and 16 mM phosphate mix Na_2_HPO_4_/KH_2_PO_4_. The reaction was stopped by boiling for 1 min and the supernatant was assayed in a 500 µl reaction mixture: 0.1 M HEPES (pH 7.6), 4 mM MgCl_2,_ and 1.5 mg ml^-1^ NADP. The resulting Glc1P was determined spectrophotometrically by calculating the change in absorbance at 340 nm after addition of 3 units PGM (EC 5.4.2.2) and 3 units G6PDH.

The extraction and assay of PEPC was based on the method described by Borland and Griffiths (1997). About 200 mg leaf material was homogenized in 1 ml extraction buffer at 4°C containing: 200 mM Tris-HCl (pH 8.0), 2 mM EDTA, 1 mM dithiothreitol (DTT), 2% (w/v) polyethylene glycol (PEG) 20,000, 1 mM benzamidine, and 10 mM malic acid with 240 mM NaHCO_3_. The homogenate was centrifuged for 2 min at 16,200 g. The extract was then desalted by passing twice through a 0.5-ml column of Sephadex G-25, equilibrated with 100 mM Tris-HCl (pH 7.5 at 4°C), 1 mM DTT, 1 mM benzamidine, and 5% (w/v) glycerol. The Ki of PEPC for malic acid was estimated using different malic acid concentrations (0.25, 0.5, 2, 8, 16 mM) in a reaction mix (500 µl) containing: 65 mM Tris-HCl (pH 7.5), 5 mM MgCl_2_, 0.2 mM NADH, 10 mM NaHCO_3,_ and 2.5 mM PEP. Production of oxaloacetate by PEPC was coupled to oxidation of NADH by the high endogenous NAD-dependent MDH activity in the extracts. The reaction was initiated by the addition of 50 µl of extract and change in absorbance at 340 nm was measured for 4 min at 25°C. Preliminary experiments confirmed a linear decrease of NADH for during at least 6 min.

### Data Analyses

Where appropriate, data were analyzed using the statistical software package IBM SPSS Statistics V23. Before carrying out statistical tests, normality of the data was checked by means of the Kolmogorov-Smirnoff statistic (p > 0.05). Means are compared by independent sample t-test (α = 0.05). All replicates considered in our study were independent biological replicates originating from different plants.

## Results

### Nocturnal CO_2_ Uptake and Starch Degradation

**Figure 1:**
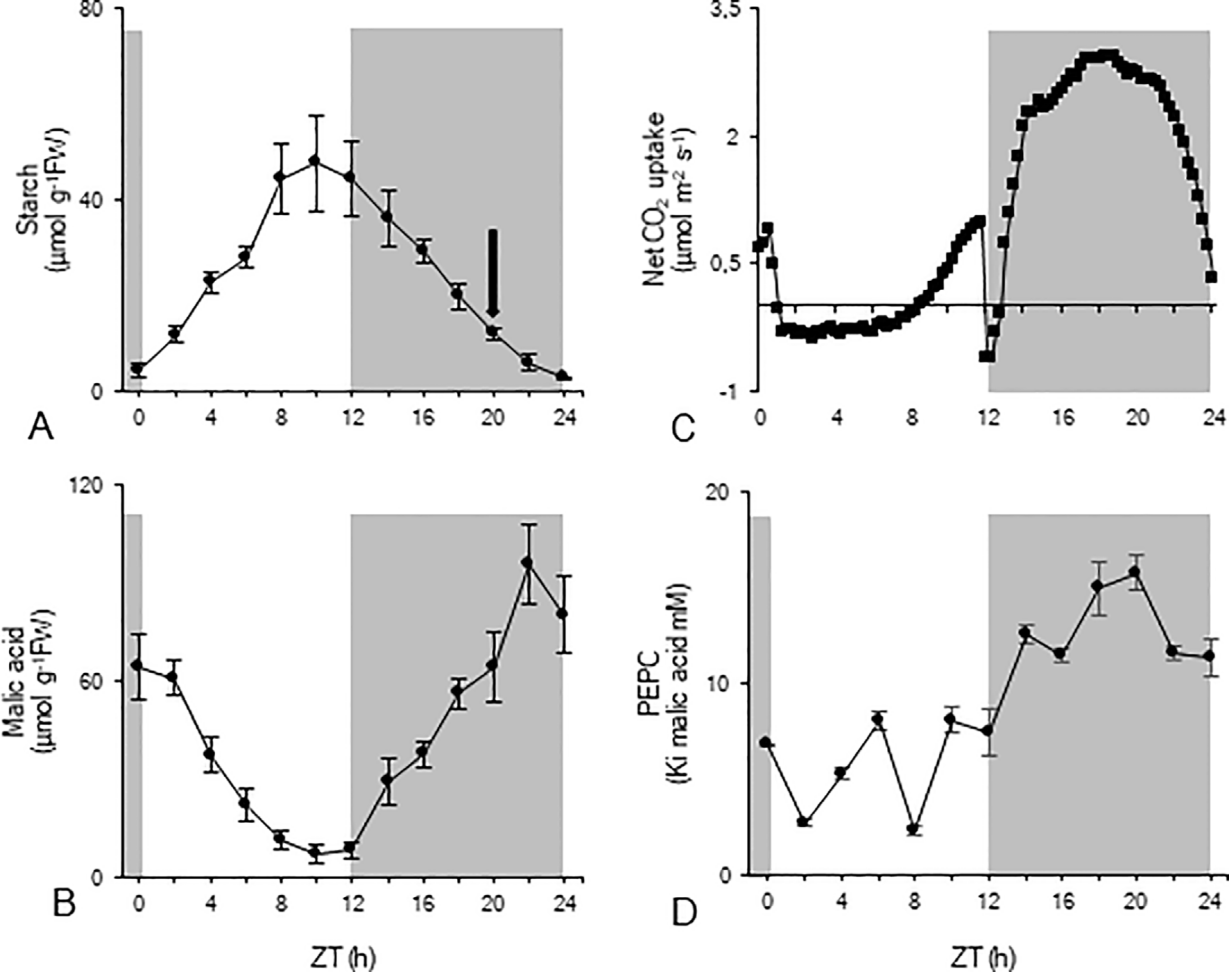
Diel patterns of starch (µmol g^-1^FW, **A**), malic acid (µmol g^-1^FW, **B**), leaf gas exchange (µmol m^-2^ s^-1^, **C**), and Ki of PEPC for malic acid (mM, **D**) for young fully developed leaves of *Phalaenopsis* ‘Edessa’. The black arrow in panel **(A)** indicates the division of the nocturnal period based on the calculated starch degradation rates. The dark period is indicated in grey. Data are means ± SD (n = 5 for **A**, **B** and **D**; n = 3 for **C**).

To investigate nocturnal starch degradation and the associated enzyme activities in more detail, nocturnal starch breakdown was divided into two distinct periods based on the calculated rates of breakdown (**Figure 1A**). During the first period of the night (from ZT12 to ZT20), nocturnal degradation of starch proceeded nearly linearly (y = -20.21 x + 131.16; R^2^ = 0.998) with a mean rate of 67 ± 13 nmol g^-1^FW min^-1^, providing 32 ± 7 µmol Glc eq. g^-1^FW which is equivalent to the provision of 64 µmol g^-1^FW of PEP. Moving closer to dawn, from ZT20 to ZT24, nocturnal starch degradation was also linear but (y = -11.48 x +40.10; R^2^ = 0.974) proceeded with a significantly lower rate of 38 ± 6 nmol g^-1^FW min^-1^ and provided 9 ± 2 µmol Glc eq. g^-1^FW which is equivalent to 18 µmol g^-1^FW of PEP. In each of the considered periods, starch degradation provided enough substrate to sustain nocturnal malic acid synthesis (**Figure 1B**) with an initial accumulation of 56 ± 10 µmol g^-1^FW followed by another 20 ± 7 µmol g^-1^FW at the end of the night. In accordance, nocturnal CO_2_ uptake (**Figure 1C**) was about 96 ± 8 mmol m^-2^ for the first nocturnal period and 35 ± 9 mmol m^-2^ for the second period. The pattern of leaf gas exchange showed increasing CO_2_ uptake during the first three hours (ZT12 – ZT15) of the dark period whilst a decreasing CO_2_ assimilation rate was noticed during the final three hours (ZT21 – ZT24) of the dark period (**Figure 1C**). Measurements of Ki PEPC for malic acid fluctuated during the day around a low mean value of 6 ± 3 mM. During the first period of the night (from ZT12 to ZT20) Ki values strongly increased to a maximum value of 16 ± 1 mM (ZT20), and slightly decreased during the second period of the night to 11 ± 1 mM (ZT24) (**Figure 1D**).

### Diel Patterns of Sugars and Sugar Intermediates

**Figure 2:**
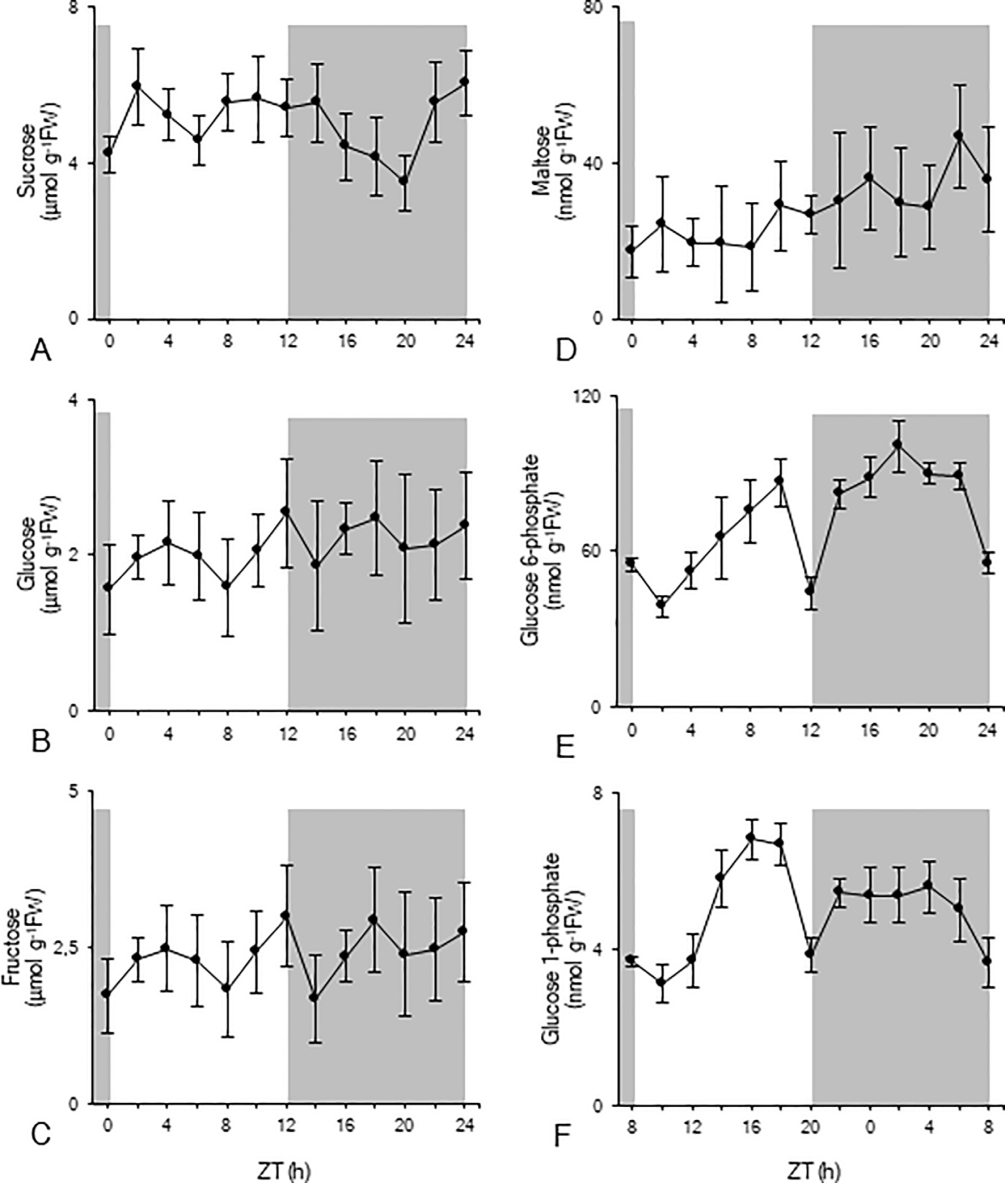
Diel patterns of sucrose (µmol g^-1^FW, **A**), glucose (µmol g^-1^FW, **B**), fructose (µmol g^-1^FW, **C**), maltose (nmol g^-1^FW, **D**), glucose 6-phosphate (nmol g^-1^FW, **E**), and glucose 1-phosphate (nmol g^-1^FW, **F**) for young fully developed leaves of *Phalaenopsis* ‘Edessa’. The dark period is indicated in grey. Data are means ± SD (n = 5).

The diel pattern of sucrose showed a rather stable diurnal phase followed by a significant (p < 0.05) nocturnal decrease to a minimum value of 3 ± 1 µmol g^-1^FW, before rising again in the later part of the night to predusk levels (**Figure 2A**). Glucose and fructose concentrations were relatively stable during the diel cycle and fluctuated around 2 ± 1 µmol g^-1^FW (**Figures 2B, C**). Leaf maltose contents remained rather stable during the photoperiod, varying around 22 ± 10 nmol g^-1^FW, and doubled during the dark period, reaching a peak value of 47 ± 13 nmol g^-1^FW (ZT22) (**Figure 2D**). Glc6P (**Figure 2E**) concentrations generally increased during daytime until a dramatic decrease at the day-night transition (ZT12) from 86 ± 9 nmol g^-1^FW to 44 ± 6 nmol g^-1^FW. After two hours in the dark, values restored again to 82 ± 6 nmol g^-1^FW (ZT14) and remained relatively stable during the remainder of the night, until a strong decrease during the last two hours of the dark period (ZT22 –ZT24). A similar diel pattern was observed for Glc1P (**Figure 2F**). During daytime concentrations generally increased, until a dramatic decrease at the day-night transition (ZT12) from 7 ± 1 nmol g^-1^FW to 4 ± 1 nmol g^-1^FW. After two hours in the dark, values restored to 5 ± 1 nmol g^-1^FW, and remained relatively stable during the remainder of the night, followed by a gradual decrease during the last four hours of the dark period (ZT20 – ZT24).

### Diel Activity Patterns of Enzymes Implicated in Starch Degradation

**Figure 3:**
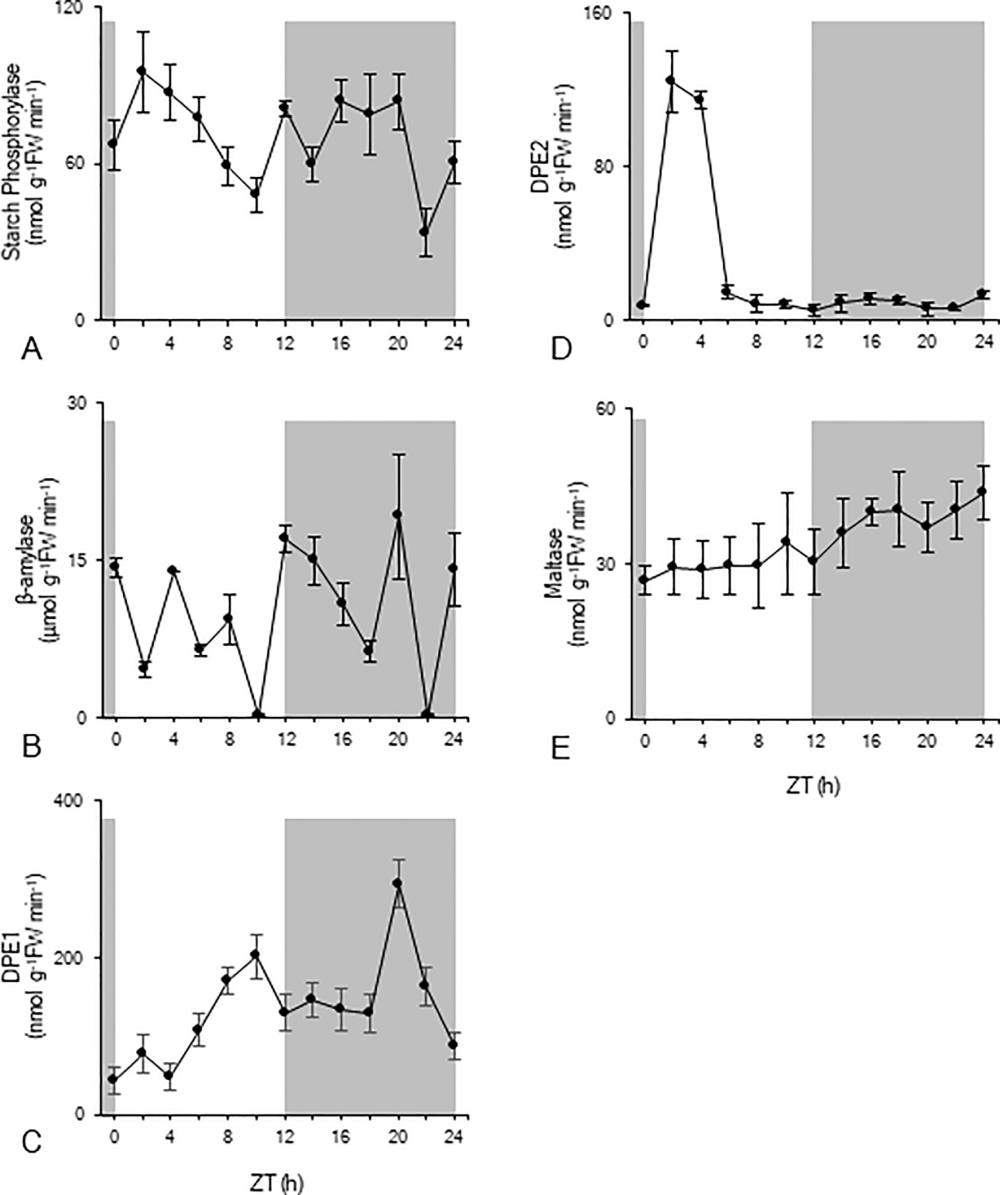
Diel enzyme activity of starch phosphorylase (nmol g^-1^FW min^-1^, **A**), β-amylase (µmol g^-1^FW min^-1^, **B**), DPE1 (nmol g^-1^FW min^-1^, **C**), DPE2 (nmol g^-1^FW min^-1^, **D**), and maltase (nmol g^-1^FW min^-1^, **E**) for young fully developed leaves of *Phalaenopsis* ‘Edessa’. The dark period is indicated in grey. Data are means ± SD (n = 5).

In order to gain more insight into the nocturnal starch degradation process, total extractable activities of a range of enzymes implicated in starch degradation were measured over the complete diel cycle (**Figure 3**). Starch phosphorylase activity significantly (p < 0.05) decreased during daytime from 95 ± 15 nmol g^-1^FW min^-1^ to 48 ± 6 nmol g^-1^FW min^-1^ (**Figure 3A**). Compared to these daytime values, nocturnal starch phosphorylase activity was higher, fluctuating around a mean of 68 ± 8 nmol g^-1^FW min^-1^ (**Figure 3A**). β-amylase showed the highest activity of all enzymes but without any clear pattern over the 24-h cycle (**Figure 3B**). Heavily fluctuating activities between 0.21 ± 0.01 µmol g^-1^FW min^-1^ and 19 ± 6 µmol g^-1^FW min^-1^ were observed during both day and night. The chloroplastic disproportionating enzyme (DPE1) showed an increasing activity during daytime from 44 ± 16 nmol g^-1^FW min^-1^ (ZT0) to 200 ± 28 nmol g^-1^FW min^-1^ (ZT10) (**Figure 3C**). Except for the observed high activity at ZT20 (317 ± 60 nmol g^-1^FW min^-1^), nocturnal DPE1 activity was quite stable around a mean value of 156 ± 12 nmol g^-1^FW min^-1^. For cytosolic disproportionating enzyme (DPE2) high activities were observed during the photoperiod at ZT2 and ZT4, i.e. 124 ± 16 nmol g^-1^FW min^-1^ and 112 ± 4 nmol g^-1^FW min^-1^ respectively. However, during the remainder of the day and night, the enzyme showed an overall low activity of only 8 ± 4 nmol g^-1^FW min^-1^ (**Figure 3D**). Maltase activity remained relatively stable during the photoperiod with a mean activity of 28 ± 8 nmol g^-1^FW min^-1^ (**Figure 3E**). During the dark period, maltase activity significantly (p < 0.05) increased from a minimum activity of 30 ± 6 nmol g^-1^FW min^-1^ (ZT12) to a maximum activity of 44 ± 5 nmol g^-1^FW min^-1^ (ZT24).

### Nocturnal Starch Degradation and Calculated Enzymatic Capacities

The nocturnal *in vitro* activities of the enzymes involved in starch degradation (starch phosphorylase, β-amylase, DPE1, DPE2, and maltase) were considered relative to the upper and lower limits of the observed starch degradation rate i.e. 67 ± 13 nmol g^-1^FW min^-1^ from ZT12-ZT20 and 38 ± 6 nmol g^-1^FW min^-1^ from ZT20-ZT24. Registered minimum, maximum, and mean activities were all considered for each enzyme in comparison with the calculated nocturnal starch degradation rate and symbols were used to indicate whether enzymatic capacity was theoretically sufficient (√) or insufficient (X) to meet the observed starch degradation rates (**Table 1**). These considerations indicated that even the lowest recorded values of either β-amylase or DPE1 activity were significantly higher (p < 0.05) than the calculated nocturnal starch degradation rates for both distinct periods. Exceeding starch degradation rates by multiples of two for DPE1 to even 6–80 for β-amylase, these particular enzymes were not rate-limiting at all in the starch degradation process. In contrast even the maximum values of DPE2 activity were far below (p < 0.05) the calculated nocturnal starch degradation rates for both periods and could only account for about 15% of the calculated starch degradation. Whilst maltase activity was not limiting starch degradation during the second period (p > 0.05) it could only account for about 50% of the calculated starch degradation during the first period of the night (p < 0.05). Considering the summed activity of DPE2 and maltase, it could still only account for about 65% of the calculated starch degradation during the first period of the night (p < 0.05). For both considered periods the calculated starch degradation rates perfectly matched the observed min-max range for starch phosphorylase activities. Only during the first nocturnal period minimum observed activities of starch phosphorylase might potentially limit starch degradation accounting for about 75% of starch breakdown (p < 0.05).

**Table 1:** Comparison between nocturnal starch degradation rate (nmol g^-1^FW min^-1^) and calculated nocturnal activity (minimum, maximum and mean) of starch degradative enzymes (nmol g^-1^FW min^-1^) in young fully developed leaves of *Phalaenopsis* ‘Edessa’, for both nocturnal periods.

Nocturnal starch degradation rate (nmol g^-1^FW min^-1^)		Period 1 67 ± 13		Period 2 38 ± 6	
		Activity (nmol g^-1^FW min^-1^)			
Starch phosphorylase	MIN	52 ± 11 *	X	33 ± 8	√
	MAX	84 ± 8 *	√	86 ± 11 *	√
	MEAN	76 ± 13	√	59 ± 23	√
β-amylase	MIN	6301 ± 1041 *	√	259 ± 48 *	√
	MAX	19167 ± 6017 *	√	19167 ± 6017 *	√
	MEAN	13661 ± 5138 *	√	11176 ± 9788 *	√
DPE1	MIN	129 ± 24 *	√	87 ± 17 *	√
	MAX	316 ± 59 *	√	316 ± 59 *	√
	MEAN	171 ± 82 *	√	186 ± 118 *	√
DPE2	MIN	4 ± 3 *	X	5 ± 3 *	X
	MAX	11 ± 3 *	X	13 ± 2 *	X
	MEAN	8 ± 3 *	X	8 ± 4 *	X
Maltase	MIN	30 ± 6 *	X	37 ± 5	√
	MAX	41 ± 7 *	X	44 ± 5	√
	MEAN	37 ± 4 *	X	40 ± 3	√
DPE2 + Maltase	MIN	36 ± 4 *	X	40 ± 8	√
	MAX	52 ± 4 *	X	56 ± 8	√
	MEAN	44 ± 8 *	X	48 ± 8	√

To investigate nocturnal starch degradation and the associated enzyme activities in more detail, the nocturnal part was divided into two distinct periods i.e. Period 1 from ZT12 to ZT20 and Period 2 from ZT20 to ZT24. Symbols are used to indicate if the enzymatic capacity is sufficient (√) of insufficient (X) to accommodate the observed starch degradation.

Data are means ± SD (n = 5) and asteriks indicate significant differences between the calculated enzymatic activity and the nocturnal starch degradation rate of the same period at *P < 0.05 according to the independent sample t-test.

### Starch Degradative Enzyme Activities in *Phalaenopsis* Match With Those in *Kalanchoë* But Are Clearly Different From Published Arabidopsis Values

To strengthen our study, we further compared our mean values of enzyme activities from *Phalaenopsis* with either a negative control C3 photosynthesis species such as Arabidopsis (based on published data) and a positive control CAM species such as *K. fedtschenkoi* (**Table 2**; to allow direct comparison the *Phalaenopsis* values were recalculated to mass basis). The data clearly indicated that starch phosphorylase activity in the model CAM plant *K. fedtschenkoi* could also easily accommodate the observed starch degradation whilst DPE2 + maltase fell short. These observations strongly contradicted with Arabidopsis showing a completely different situation with high DPE2 activity, supporting starch hydrolysis, and insufficient starch phosphorylase activity. Moreover, high DPE2 and low starch phosphorylase activities have also been reported by Borland et al. (2016) for Arabidopsis whilst the reverse was true for *M. crystallinum* (in the CAM mode) under identical growth conditions.

**Table 2:** Comparison of nocturnal starch degradation rate (nmol g^-1^FW min^-1^) and mean activities of starch degradative enzymes (nmol g^-1^FW min^-1^) in leaves of *Phalaenopsis* ‘Edessa’, *Kalanchoë fedtschenkoi* and *Arabidopsis thaliana*.

	CAM	CAM reference	C3 reference
	*Phalaenopsis* ‘Edessa’	*K. fedtschenkoi*	Arabidopsis
Starch phosphorylase	**76 ± 12**	**209 ± 38**	**40 ± 7**
β-amylase	13674 ± 5133	5293 ± 376	140 ± 27
DPE1	132 ± 8	1760 ± 209	273 ± 28
DPE2	**8 ± 4**	**31 ± 3**	**146 ± 3**
Maltase	**36 ± 4**	**43 ± 2**	**20 ± 2**
Nocturnal starch breakdown	68 ± 12	93 ± 11	∼92

Data are means ± SD (n = 5). Values for Arabidopsis originate from Chia et al. (2004). Different colors have been used to indicate whether the relevant enzymatic capacities in either starch phosphorolysis (starch phosphorylase) or hydrolysis (DPE2 + maltase) are adequate (green) or inadequate (red) to accommodate the observed starch degradation.

## Discussion

### Regulation of Nocturnal Starch Degradation to Avoid Premature Exhaustion of Carbohydrate

Carbohydrate availability represents a key limiting factor for CAM productivity, which makes the diel breakdown and resynthesis of a transient pool of carbohydrate a central requirement for CAM homeostasis (Borland and Dodd, 2002; Cushman et al., 2008). To account for the observed gradual decrease in starch degradation rate towards the end of the night in the leaves of *Phalaenopsis* “Edessa” we considered two starch degradation rates in our study i.e. (1) 67 ± 13 nmol g^-1^FW min^-1^ from ZT12 to ZT20 and (2) 38 ± 6 nmol g^-1^FW min^-1^ from ZT20 to ZT24 (**Figure 1**). Avoiding premature exhaustion of reserve carbohydrate availability before the onset of dawn is consistent with observations in the C3 plant Arabidopsis where the rate of degradation of transitory starch during the night was enhanced or declined following either shortened or lengthened photoperiods (Lu et al., 2005; Graf et al., 2010). The exact physiology behind the mechanisms that allow the plant to match starch degradation to its needs and the length of night are still exciting topics of current investigation (Fernandez et al., 2017; Flis et al., 2019). In *Phalaenopsis* “Edessa” nocturnal starch breakdown during the 4 final hours was reduced with ca. 33% and accompanied by a threefold reduction in nocturnal CO_2_ fixation and malic acid accumulation (**Figure 1**). It is very reasonable that a reduced availability of carbon skeletons for PEP synthesis at the end of the night will lead to both the reduced rate and total amount of CO_2_ fixed by PEPC in CAM plants. Carbohydrate limited gas exchange towards the end of the dark period is also consistent with the fact that the Ki of PEPC for malic acid showed an upward trend during the major part of the night but started to decrease during the 4 final hours (**Figure 1**). High values of Ki are indicative of phosphorylated PEPC through PEPC-kinase action, rendering PEPC less sensitive for malic acid inhibition (Nimmo et al., 1986; Borland and Griffiths, 1997; Borland et al., 1999). Expression of the kinase itself is not likely to bring about less phosphorylation towards dawn as similar declines in Phase-I CO_2_ assimilation well before the onset of dawn have been noticed in other CAM plants such as *M. crystallinum*, *K. daigremontiana* and *Clusia rosea*, whilst transcript abundance of PEPC kinase remained unaffected during the second nocturnal period (Borland et al., 1998; Borland et al., 1999; Dodd et al., 2003).

### The Phosphorolytic Pathway Can Accommodate the Observed Nocturnal Starch Degradation

Both the phosphorolytic and hydrolytic pathways of starch degradation are assumed to be deployed by CAM species to balance the sink demands of CAM with growth and maintenance (Borland et al., 2016). This strongly contrasts with C3 plants where starch is degraded at night *via* the hydrolytic route (Niittylä et al., 2004; Weise et al., 2004; Stitt and Zeeman, 2012), whilst phosphorolytic starch degradation mainly provides substrate for internal chloroplast metabolism (Zeeman et al., 2004; Weise et al., 2006). To increase our insights in the possible relative contributions of either pathway in the process of CAM starch degradation we compared minimum and maximum enzyme activities of a range of enzymes during the night with different rates of observed nocturnal starch degradation in leaves of the CAM orchid *Phalaenopsis* “Edessa” (**Table 1**). Starch degradation is a complex process that requires different enzymes working in sequence and/or concert and attacking different substrates. Chloroplastic starch phosphorylase could potentially account for nearly all starch degradation during the whole night with mean activities matching exactly the calculated starch breakdown and as such avoiding premature exhaustion of starch. In accordance with a decreased starch degradation during the latter part of the nocturnal period, starch phosphorylase activity also slowed down towards dawn. High levels of Glc6P were present during the major part of the night, almost three times higher compared to the observed levels of maltose (i.e. 89 ± 7 nmol g^-1^FW and 31 ± 4 nmol g^-1^FW respectively). This is consistent with an important role for starch phosphorylase in starch degradation, since Glc6P is the major export product from phosphorolytic starch degradation. Glc6P is not only considered an allosteric activator of PEPC but can also easily be converted to PEP in the cytosol providing ATP by substrate-level phosphorylation and offering energetic advantages (Holtum et al., 2005; Cheung et al., 2014). According to recently developed diel flux balance models for CAM this would make a marked difference to help offset the energetic costs of running the CAM cycle (Shameer et al., 2018).

### Hydrolytic Starch Degradation Is Limited by DPE2 and Maltase Activity

The other starch degrading enzymes residing in the chloroplast and belonging to the hydrolytic starch conversion pathway, i.e. β-amylase and DPE1, showed *in vitro* activities far exceeding those required for *in vivo* starch degradation by multiples of 2 to 80 (**Table 1**). Those high activities could potentially lead to complete exhaustion well before the end of the dark period without any further regulation. However, further processing of maltose, generated by degradation of starch *via* β-amylase, in the cytosol is dependent on either cytosolic disproportionating enzyme (DPE2) or maltase. DPE2 showed only very low nocturnal activities in *Phalaenopsis* and could theoretically meet only about 15% of the observed starch breakdown. Low DPE2 activities have also been reported in *M. crystallinum* and have previously been hypothesized to potentially represent a possible bottleneck in the hydrolytic starch degradation process (Borland et al., 2016). This is in marked contrast with the critical importance of DPE2 in C3 plants such as Arabidopsis where DPE2 activity, which is about 20 times higher than in *Phalaenopsis*, can easily account for the measured starch degradation (**Table 2**). Whilst maltase activities in Arabidopsis were considered of minimal importance and about seven-fold lower compared to DPE2 activity (**Table 2**), our measurements showed a four-fold higher mean maltase activity than DPE2 during the night in *Phalaenopsis* “Edessa,” which was still insufficient to accommodate nocturnal starch degradation. However, it can be assumed that both enzymes, DPE2 and maltase, can act in concert to accommodate maltose processing in the cytosol. Considering the sum of both enzymatic activities, the calculated minimum, maximum, and mean activity were still significantly lower (p < 0.05) than required to account for the observed nocturnal starch degradation (**Table 1**). In the model CAM plant *K. fedtschenkoi* DPE2 activity was higher but still insufficient to account for the measured starch breakdown in concert with maltase, whilst β-amylase was not rate limiting (**Table 2**). The bottleneck effect of the maltose processing enzymes in regulating β-amylase mediated starch breakdown was also depicted by a significant nocturnal accumulation (p < 0.05) of its substrate, maltose, from 27 ± 5 nmol g^-1^FW (ZT12) to 47 ± 13 nmol g^-1^FW (ZT22) in *Phalaenopsis* leaves.

In conclusion we compared *in vivo* nocturnal starch degradation and *in vitro* enzyme activity measurements during the whole night in leaves of the CAM orchid *Phalaenopsis* “Edessa” to shed more light into the interplay of the phosphorolytic and hydrolytic processes of starch degradation in CAM. We showed that the phosphorolytic pathway is the major route to accommodate nocturnal starch degradation and that measured activities of starch phosphorylase perfectly matched calculated starch degradation rates in order to avoid premature exhaustion of starch reserves before dawn. The hydrolytic pathway seemed hampered in starch processing not by β-amylase but through insufficient catalytic capacity of both DPE2 and maltase. These considerations were further corroborated by measurements of enzyme activities in the CAM model plant *Kalanchoë fedtschenkoi* and strongly contradict with the situation in the C3 plant Arabidopsis. The data support the view that the phosphorolytic pathway might be the main route of starch degradation in CAM to provide substrate for PEP with additional hydrolytic starch breakdown to accommodate mainly sucrose synthesis.

## Data Availability Statement

All datasets generated for this study are included in the article/supplementary material.

## Author Contributions

NC, WV and JC proposed the conceptual framework for the study and performed the data collection and analysis. NC and MF performed the experimental analyses. NC and JC interpreted the data and wrote the manuscript.

## Funding

This research was supported by the Research Fund KU Leuven.

## Conflict of Interest

The authors declare that the research was conducted in the absence of any commercial or financial relationships that could be construed as a potential conflict of interest.
